# The Significance of Microbial Symbionts in Ecosystem Processes

**DOI:** 10.1128/mSystems.00127-19

**Published:** 2019-05-21

**Authors:** Roxanne A. Beinart

**Affiliations:** a Graduate School of Oceanography, University of Rhode Island, Narragansett, Rhode Island, USA

**Keywords:** ecosystem processes, microbial ecology, microbial physiology, symbiosis

## Abstract

It is increasingly accepted that the microbial symbionts of eukaryotes can have profound effects on host ecology and evolution. However, the relative contribution that they make directly to ecosystem processes, like energy and nutrient flows, is less explicitly acknowledged and, in many cases, only poorly constrained. Here, I explore the idea that, in some habitats, host-associated microbes may have an outsized role in ecosystem processes relative to functionally equivalent free-living microbes due to key aspects of the physiology, ecology, and evolution of symbiotic interactions. My research quantifying symbiont metabolism has shown that microbial symbionts have the potential to make a substantial impact on carbon and sulfur cycling. It is my perspective that direct measurement of symbiont activity and comparison to free-living counterparts will expand our understanding of the significance of microbial symbioses and, more broadly, the role of microbial processes in ecosystems.

## Abstract (precis)

It is increasingly accepted that the microbial symbionts of eukaryotes can have profound effects on host ecology and evolution. However, the relative contribution that they make directly to ecosystem processes, like energy and nutrient flows, is less explicitly acknowledged and, in many cases, only poorly constrained.

## PERSPECTIVE

It is increasingly accepted that the microbial symbionts of eukaryotes can have profound effects on host ecology and evolution. However, the relative contribution that host-associated microbes make directly to ecosystem processes, like biomass production and nutrient flows, is less explicitly acknowledged and, in many cases, only poorly constrained. The microbial symbionts of eukaryotes are involved in the transformation of energy and matter via their participation in carbon, nitrogen, phosphorus, sulfur, and other cycles. In this perspective, I explore the hypothesis that, in some habitats, host-associated microbes do not just participate in these ecosystem processes, but actually have the potential to play an outsized role relative to functionally equivalent free-living microbes.

### Symbiont involvement in ecosystem processes.

Host-associated microbial symbionts are critical to the conversion of inorganic carbon, nitrogen, and/or phosphorus into organic biomass. Microbial symbionts create organic material through carbon and nitrogen fixation, as well as via inorganic nitrogen and phosphorous assimilation processes. Microbial symbionts are photosynthetic primary producers in association with fungi, plants, and animals living in low-nutrient environments (1, 2). In ecosystems with high concentrations of chemical reductants, chemosynthetic bacteria are primary producers as the carbon dioxide-fixing and methane-oxidizing symbionts of marine ciliates, bivalves, crustaceans, oligochaete and polychaete worms, and gastropods (3). Bacterial symbionts fix inorganic nitrogen gas in association with photoautotrophs with high nitrogen needs (eukaryotic phytoplankton and plants), as well as with animals living in oligotrophic habitats or those subsisting on low-nitrogen food sources like wood (sponges, corals, bivalves, and insects) (1, 4). Fungal mycorrhiza and bacteria associated with plants and sponges, respectively, take up and/or assimilate inorganic nitrogen (NO_3_^−^, NH_4_^+^) and phosphorus (P_i_) (5–7). In many of these cases, the production of primary or secondary biomass is dependent on symbiont metabolism.

In addition, the microbial symbionts of eukaryotes take part in biogeochemical cycling through the remineralization of organic material and the transformation of carbon, nitrogen, phosphorous, and sulfur between inorganic forms. In gastrointestinal tracts, microbial symbionts are important decomposers of organic material, especially recalcitrant types like cellulose and lignin, back into inorganic carbon. Furthermore, the microbial symbionts found in the gastrointestinal tracts of animals and in association with anaerobic protists release significant amounts of carbon dioxide and methane from the remineralization of organic material consumed by their hosts (8–10). Host-associated microbes also participate in the transformation of methane into carbon dioxide and organic carbon; bacterial methanotrophs that are symbiotic with animals and plants oxidize methane released in the deep-sea and terrestrial ecosystems, respectively (11, 12). The microbial symbionts of plants, bivalves, polychaete tubeworms, corals, sponges, ascidians, and foraminifera participate in the cycling of inorganic nitrogen compounds, including the processes of nitrification, denitrification, and both aerobic and anaerobic ammonia oxidation (13, 14). The sulfur-oxidizing bacterial symbionts of ciliates, polychaete worms, bivalves, gastropods, and crustaceans transform hydrogen sulfide into oxidized or partially oxidized forms like sulfate and thiosulfate (3), while the sulfate-reducing symbionts of anaerobic protists and oligochaete worms are sulfate reducers that convert sulfate back into hydrogen sulfide (15, 16).

**FIG 1:**
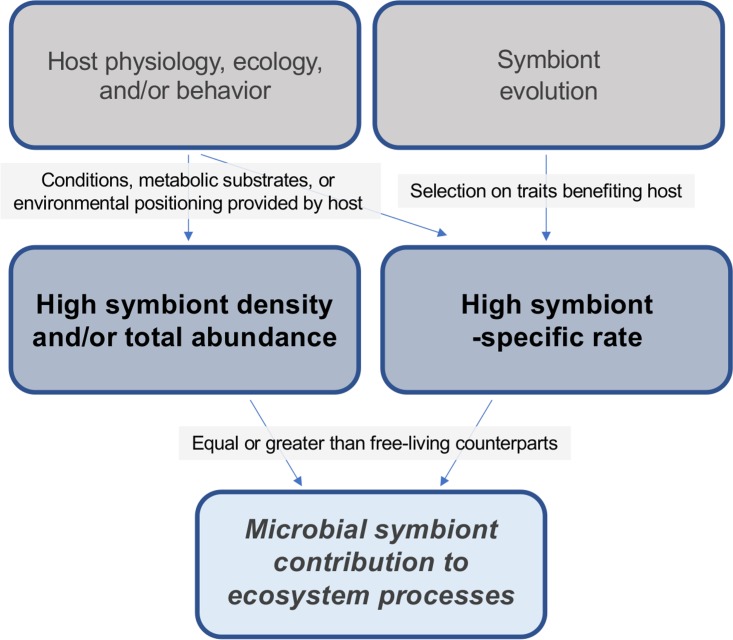
Conceptual diagram illustrating how microbial symbiont densities, total abundances, and cell-specific rates may be increased due to key physiological, ecological, and evolutionary aspects of symbiotic interactions. This, consequently, has the potential to increase their impact on ecosystem-level processes like primary production and biogeochemical cycles.

It is clear that the microbial symbionts of eukaryotes contribute to a broad range of processes relating to the flux of energy and matter. However, microbial symbionts that are involved in ecosystem processes are not simply biogeochemically active microbes that happen to be host associated. Instead, there are key aspects related to the physiology, ecology, and evolution of symbiotic partnerships that must be recognized when we consider the contribution of microbial symbionts to ecosystems. Below, I outline three arguments for why this is the case (conceptualized in Fig. 1).

**(i) Through association with a host, symbiont population density (and consequently, symbiont activity) can be large due to host protection and cultivation.** The microbial symbionts of eukaryotes can grow to high population densities or total abundances that are equivalent or larger than their free-living functional counterparts. Microbial symbionts growing in or on their hosts are often provided specialized physical space and/or protection from stressful environmental conditions, pathogens, predators, and/or competitors. There is also evidence that the immune systems of eukaryotic hosts eliminate potential competitors for their symbionts (17). Furthermore, hosts have the potential to boost symbiont growth and population density by providing substrates for metabolism, optimal abiotic conditions (e.g., anoxia), or physical positioning in the environment. Thus, based on numbers alone, symbionts may play a disproportionately important and active role in some ecosystem processes.

**(ii) Hosts provide symbionts with substrates, advantageous conditions, or optimal positioning in the environment, bolstering their relative cell-specific rates.** Eukaryotic hosts often have morphological, physiological, and/or behavioral traits that provide their symbionts with enhanced conditions for symbiont functioning. These traits may be adaptive, having evolved in response to association with a symbiont, or nonadaptive, having evolved for another purpose but boosting symbiont activity nonetheless. Symbiont cell-specific rates could be increased due to host delivery of scarce nutrients or an unusually high and/or steady concentration of a metabolic substrate. Alternatively, hosts may drive cell-specific rates through the removal of metabolic end products released by their symbionts, increasing thermodynamic favorability for otherwise unfavorable symbiont metabolisms. Further, hosts often provide an advantageous habitat that is more stable, protected, and/or distinct from the external environment, which could increase cell-specific rates by improving enzyme stability. Finally, hosts may display behaviors or morphology that optimize positioning in the environment, for example by physically spanning the interface between two environments, providing the symbiont access to resources from both environments, or by moving to ideal light, temperature, or chemical levels. Thus, by occupying a host-associated niche and without any fundamental changes to their own physiology, microbes could have higher cell-specific rates of activity.

**(iii) If symbiont function provides fitness benefits to its host, there may be selective pressure that leads to the evolution of higher cell-specific rates of this function.** Symbiont metabolisms that are advantageous to their hosts may be subject to selection that increases the cell-specific rate of that process. This could occur through the evolution of genomic changes that affect content, regulation, and expression of genes, and/or protein function and stability. Evolutionary changes in gene content could occur through the acquisition of multiple genes or pathways that have homologous function but functional differences in optimal conditions (e.g., under high or low oxygen concentrations), improving the symbiont’s ability to perform that function under a wide variety of settings, and ultimately, increasing cell-specific rates of that process. Changes could also occur that could boost cellular protein content for key processes, either through the duplication of genes or through modifications in the regulation of gene expression, that increase protein content and/or make it more consistently expressed. Finally, adaptive changes to the protein structure itself have the potential to increase cell-specific rates through increases in efficiency, stability, or other aspects of protein function. Thus, through selection on a function that provides host benefits, microbial symbionts may evolve intrinsic differences from free-living counterparts that affect their relative role in important ecosystem processes.

### Future directions.

**FIG 2:**
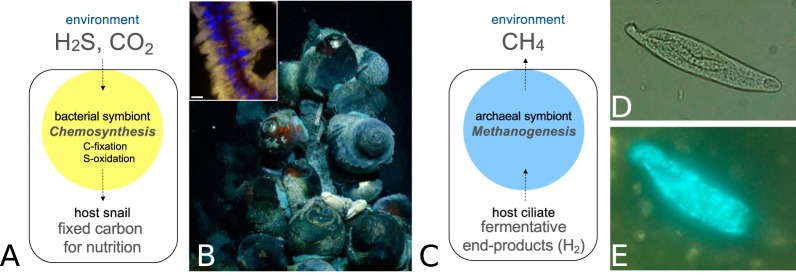
The symbiotic study systems investigated by the Beinart laboratory, emphasizing their interaction with the geochemical environment. (A) The chemosynthetic, bacterial endosymbionts of hydrothermal vent snails oxidize the hydrogen sulfide in venting fluid for the energy to fix dissolved inorganic carbon into organic carbon, which provides the bulk of the host’s nutrition. (B) *Alviniconcha* snails cling to the top of a hydrothermal vent chimney structure (courtesy of C. Fisher, Pennsylvania State University/NSF/ROV Jason/2009 © Woods Hole Oceanographic Institution, reproduced with permission). The inset micrograph shows the presence of the bacterial symbionts inside snail gill filament cells. Bacterial symbionts hybridized with fluorescent, universal bacterial probe Eub338I-III (yellow) and host nuclei stained with DAPI fluorescent DNA stain (blue) are shown. Bar, 10 μm. (C) The archaeal endosymbionts of anaerobic ciliates use host produced fermentation end products (e.g., H_2_) as the substrates for methanogenesis, making fermentation more favorable for the host, while also releasing methane to the environment. (D and E) Micrographs of a freshwater anaerobic ciliate from the genus *Heterometopus* (D) and the autofluorescence of its methanogenic, archaeal endosymbionts via excitation of coenzyme F420 (E).

Direct measurement of symbiont activity and comparison to free-living counterparts are necessary next steps in research on the significance of host-associated microbes. In particular, measurement of cell-specific rates for both symbionts and free-living counterparts, as well as investigations of the physiological, ecological, and evolutionary mechanisms that drive observed differences, are necessary to understand how symbiosis impacts microbial activity. In order to better understand the role of microbial symbionts in primary productivity and biogeochemical cycling, my research program is aimed at characterizing and experimentally quantifying symbiont activity, focusing on symbioses between animals and sulfur-oxidizing chemosynthetic bacteria, as well as those between anaerobic ciliates and methanogenic archaea (Fig. 2). Physiological experiments with both of these types of symbioses have shown that they are important in the cycling of carbon and sulfur and that they are highly active relative to communities of free-living functional counterparts. For example, the chemosynthetic bacterial symbionts associated with an average snail weighing approximately 10 g at hydrothermal vents in the western Pacific can fix as much inorganic carbon and oxidize as much hydrogen sulfide (18) as the free-living microbes in 10 liters of the surrounding seawater (19, 20). Estimates of snail-associated symbiont density will be necessary to understand whether these rates are due to the large population of symbionts in a single snail and/or whether the rates are higher on a per-cell basis. For the methanogenic symbionts of anaerobic ciliates where estimates of symbiont density are better, symbiont-specific rates of methane production are 1 to 4 orders of magnitude higher than cell-specific rates for free-living methanogens in marine and lake sediments, and approximately equivalent to cell-specific rates for methanogens in anaerobic bioreactors (10, 21; R. Beinart, unpublished data). It is not yet clear whether physiological or evolutionary mechanisms are driving these relatively high symbiont-specific rates. In both of these systems, future work in my laboratory will use an integrated combination of observational and experimental approaches to provide insight into the distribution, physiology, and activity of microbial symbioses, allowing the estimation of their contribution to biogeochemical cycles and their role in ecosystem processes. However, work on symbiont physiology beyond these two particular examples will also be critical. Given the prevalence of microbial symbioses, their participation in many fundamental nutrient and geochemical transformations, and their likely relatively high cell density and cell-specific rates, a greater understanding of symbiont physiology has the potential to advance our broad understanding of the function and significance of microbes in many ecosystems.
